# Role of Mesencephalic Astrocyte-Derived Neurotrophic Factor in Alcohol-Induced Liver Injury

**DOI:** 10.1155/2020/9034864

**Published:** 2020-07-07

**Authors:** Goma Chhetri, Yanyan Liang, Juntang Shao, Dan Han, Yi Yang, Chao Hou, Peng Wang, XiaoFang Tao, Yujun Shen, Tongcui Jiang, Lijie Feng, Yuxian Shen

**Affiliations:** ^1^School of Basic Medical Sciences, Anhui Medical University, Hefei, 230032 Anhui, China; ^2^Biopharmaceutical Research Institute, Anhui Medical University, Hefei, China

## Abstract

Consumption of alcohol in immoderate quantity induces endoplasmic reticulum (ER) stress response (alcohol-induced ER stress). Mesencephalic astrocyte-derived neurotrophic factor (MANF), an ER stress-inducible protein, works as an evolutionarily conserved regulator of systemic and liver metabolic homeostasis. In this study, the effects of MANF on alcohol-induced liver injury were explored by using hepatocyte-specific MANF-knockout mice (MANF^*Δ*Hep^) in a chronic-plus-binge alcohol feeding model. We found that alcohol feeding upregulated MANF expression and MANF^*Δ*Hep^ mice exhibited more severe liver injury with extra activated ER stress after alcohol feeding. In addition, we found that MANF deficiency activated iNOS and p65 and increased the production of NO and anti-inflammatory cytokines, which was further enhanced after alcohol treatment. Meanwhile, MANF deletion upregulated the levels of CYP2E1, 4-HNE, and MDA and downregulated the levels of GSH and SOD. These results indicate that MANF has potential protection on alcohol-induced liver injury, and the underlying mechanisms may be associated with meliorating the overactivated ER stress triggered by inflammation and oxidative stress via inhibiting and reducing NO/NF-*κ*B and CYP2E1/ROS, respectively. Therefore, MANF might be a negative regulator in alcohol-induced ER stress and participate in the crosstalk between the NF-*κ*B pathway and oxidative stress in the liver. *Conclusions*. This study identifies a specific role of MANF in alcohol-induced liver injury, which may provide a new approach for the treatment of ALI.

## 1. Introduction

Alcoholic liver disease (ALD) is one of the global health issues and has received growing attention. Ethanol-induced liver injury is largely influenced by drinking patterns, dietary options, and obesity, as well as by environmental factors [1]. ALD is composed of various forms of liver lesions, including hepatic steatosis, fibrosis, and cirrhosis. Although patients with milder ALD forms (isolated fatty liver) might be benefited from simple abstinence, patients with alcoholic hepatitis, the most severe ALD, are currently given limited therapeutic options other than short exposure to corticoids. Therefore, it is of great importance to understand the key elements involved in the progression of ALD [2–4].

Alcohol induces liver injury through several mechanisms, including inflammation, oxidative stress, and ER stress [5]. Hepatic biotransformation of ethanol into acetaldehyde mainly involves alcohol dehydrogenase (ALH) and cytochrome P450 2E1 (CYP2E1). Both oxidative metabolic pathways of ethanol lead to the production of reactive oxygen species (ROS). ROS could be removed efficiently by an endogenous protective system, including superoxide dismutase (SOD) and antioxidant GSH [6]. However, chronic alcohol consumption impairs this protective system by decreasing enzyme activity and by antioxidant depletion. As a result, abnormally elevated ROS level leads to oxidative stress and subsequent cell damage. ROS also triggers inflammatory responses and cytokine production, which mediate the adverse effects of ethanol on multiple organs [7]. Additionally, ROS induces ER stress by disturbing the redox balance and by interfering with the proper folding of the protein in the ER [8]. Alcohol-induced cell death is also associated with ER stress. Alcohol and its metabolites (e.g., acetaldehyde) are highly reactive in modifying protein structures or forming protein adducts, which leads to the accumulation of misfolded proteins and therefore triggers unfolded protein responses (UPR).

Mesencephalic astrocyte-derived neurotrophic factor (MANF) is localized in the ER lumen and is also a secretory protein. It was isolated from the conditioned medium of rat ventral mesencephalic cell line 1 (VMCL1) with selective protection of dopaminergic neurons in vitro [9–11]. MANF is an ER stress-responsive protein and plays a vital role in cell protection against ER stress-induced cell damage. ER stress induces MANF expression upon the ER inducer thapsigargin or tunicamycin. We have reported that Hela cells with MANF knockdown by siRNA showed decreased cell tolerance to ER stress-induced apoptosis [9]. On the other hand, MANF overexpression or recombinant MANF supplement in vitro alleviated ER stress and promotes cell survival [12, 13]. In a rat focal ischemia model, recombinant human MANF significantly inhibited ischemia-induced increase in BiP, p-IRE1, and XBP1s and prevented neuron loss in a rat focal brain [14]. Recent studies have demonstrated that MANF plays a significant role in immune cell activation, inflammation regulation, retina repair, and hepatic metabolism homeostasis [15–18]. MANF appears to modulate inflammatory pathways that are common to many age-related diseases. Aging has been reported to regulate some of the factors causing ER stress. It has an important impact on the proteostasis network, which means that aging cells may have diminished capacity to carry out protein transcription, translation, folding, and degradation properly [19, 20]. Aging cells also have been shown to have reduced expression levels of ER-related proteins, along with protein chaperones (such as PDI and BIP), which are normally responsible for regulating misfolded proteins towards degradation and for assisting in proper protein folding [21–23]. We have found that splenic macrophages lacking MANF were more prone to display proinflammatory phenotypes (M1-type) upon activation [15]. Recently, we reported that in comparison to liver cancer tissues in HCC patients, the MANF level was higher in the adjacent noncancer tissues of the same patient. MANF also acts as a cancer suppressor by inhibiting the NF-*κ*B/Snail signal pathway in HCC progression [16]. However, how hepatic MANF expression is affected by alcohol exposure and whether MANF can contribute to the hepatocyte survival in alcohol-induced liver injury remain unknown.

In this study, we used a hepatocyte-specific MANF-knockout mouse model to investigate the role of hepatocyte-derived MANF in ALI. We observed that MANF expression was upregulated in liver tissue of mice after chronic-plus-binge alcohol feeding, and MANF^*Δ*Hep^ mice were more susceptible to alcohol-induced liver injury. Based on the observed results, we propose that hepatocyte-derived MANF might exert protective effects in ALI by alleviating ethanol-induced ER stress, oxidative stress, and inflammation.

## 2. Methods and Materials

### 2.1. Animals

All animal studies were conducted in full compliance following the guidelines of the Care and Use of Laboratory Animals of the Ministry of Sciences and Technology. Anhui Medical University approved the experimental animal protocol. The NIAAA (National Institute on Alcohol Abuse and Alcoholism) model was applied in ethanol feeding studies. Hepatocyte-specific MANF-knockout (MANF^*Δ*Hep^) mice were constructed as to the previous description [16]. Female wild-type C57BL/6J mice (8-10 weeks old) and MANF^*Δ*Hep^ mice were housed in pairs per cage in a temperature-controlled room at 25°C with a 12-hour light-dark cycle. Mice were fed with controlled Lieber-DeCarli diet to acclimatize the mice to the liquid diet for the first five days.

During the following 10 days, mice were fed with free access to ethanol Lieber-DeCarli diet (Trophic Animal Feed High-Tech Co. Ltd., China), which contained 5% (vol/vol) ethanol. Mice in the control group were pair-fed with an isocaloric controlled diet. In the early morning on day 11, a single dose of alcohol (5 g/kg body weight) or isocaloric dextrin maltose was administered via oral gavage to mice in ethanol- and pair-fed groups, respectively. Mice were euthanized 9 hours later for tissue collection. Daily alcohol consumption by MANF^*Δ*Hep^ and WT mice was measured, and they show comparable alcohol intake levels [24].

### 2.2. Biochemical Assays

Alanine aminotransferase (ALT) activity, aspartate aminotransferase (AST) activity, superoxide dismutase (SOD) activity, malondialdehyde (MDA) level, glutathione (GSH) level, and nitric oxide (NO) and triglyceride (TG) levels were measured using commercial assay kits according to the manufacturer's manuals (Nanjing Jiancheng Bioengineering Institute, Jiangsu, China).

### 2.3. Immunohistochemical Staining

Immunohistochemistry was performed on liver sections as previously described [25]. Briefly, formalin-fixed, paraffin-embedded liver sections were dewaxed and rehydrated. Antigen retrieval was conducted by using pH 6.0 citrate buffer or proteinase K treatment. Furthermore, endogenous peroxidase activity was quenched in 0.3% H_2_O_2_. Sections were incubated with primary antibodies overnight at 4°C. Appropriate biotinylated secondary antibodies were incubated with sections for 1 h at 37°C. Then, samples were incubated with horseradish peroxidase-conjugated streptavidin for 15 min at 37°C. Immunostaining was developed with the DAB Peroxidase Substrate Kit. Moreover, sections were counterstained with hematoxylin. Details on primary antibodies are listed in Supplementary Table 1. Images were acquired using Olympus Microscope BX53 and cellSens Standard software. Average staining areas were calculated using ImageJ software based on 10 randomly selected microscopic fields.

### 2.4. Western Blot

Liver tissues were homogenized in lysis buffer, and supernatants were collected after centrifugation (15,000 g, 4°C for 15 min). Aliquots containing an equal amount of tissue proteins were subjected to 12% SDS-PAGE, followed by western blot against primary antibodies. The primary antibodies used in these are enumerated in Supplementary Table 2. Band densities were quantified using Chemi Analysis software and ImageJ.

### 2.5. Real-Time qPCR

Total RNA from liver tissues was extracted using the TRIzol reagent (Invitrogen). RNA was further reverse transcribed into cDNA. Target gene transcripts were quantified by real-time quantitative PCR with SYBR green using the ABI7500 real-time PCR detection system (Applied Biosystems by Thermo Fisher Scientific). The primer sequences used for RT-qPCR are listed in Supplementary Table 3.

### 2.6. Statistical Analysis

For statistical analysis, data were expressed as means ± standard error of the mean (SEM). Results were analyzed using one-way ANOVA or two-tailed Student's *t*-test with GraphPad Prism 5.0 software. Differences with values of *P* < 0.05 were considered statistically significant.

## 3. Results

### 3.1. Upregulation of MANF in Alcoholic Liver Injury

**Figure 1:**
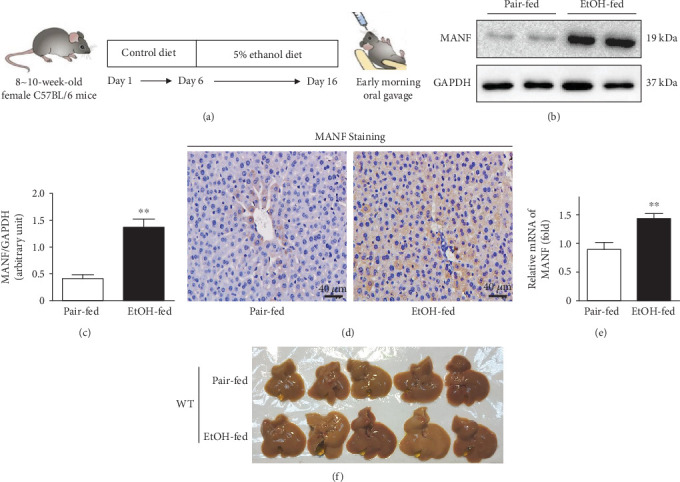
Upregulation of MANF in EtOH-fed mice. (a) The feeding schedule of the Gao-Binge model. (b) Western blot analysis of MANF protein level in WT mouse livers. GAPDH was used as a loading control. (c) The quantitative data of (b). ^∗∗^*P* < 0.01, compared with the pair-fed group. (d) Immunostaining for MANF in paraffin sections of WT mouse livers. Scale bar = 40 *μ*m. (e) Relative mRNA level of MANF in WT mouse livers. (f) Gross examination of the WT liver. ^∗∗^*P* < 0.01, compared with the pair-fed group. EtOH: ethanol.

It has been reported that female mice are more susceptible to alcohol-induced liver injury than male mice [24]. Therefore, we used female mice in this study. The Gao-Binge (a chronic-plus-binge alcohol feeding) model was used to mimic common drinking patterns among the patients with ALD [24]. Mice in the ethanol group were following the feeding schedule depicted in Figure 1(a). To examine whether alcohol-induced liver injury influences the hepatic expression of MANF, we detected the levels of MANF protein and mRNA in the liver tissues from the model mice. Hepatic MANF protein level was significantly increased in the model mice compared with the pair-fed mice by using the assays of western blotting and immunohistochemical staining (Figures 1(b)–1(d)). Similarly, the mRNA level of MANF was upregulated after chronic-plus-binge alcohol exposure (Figure 1(e)). Our data also show that the volume of the livers became bigger after ethanol exposure in the WT group (Figure 1(f)).

### 3.2. MANF^*Δ*Hep^ Mice Are More Susceptible to Alcoholic Liver Injury

**Figure 2:**
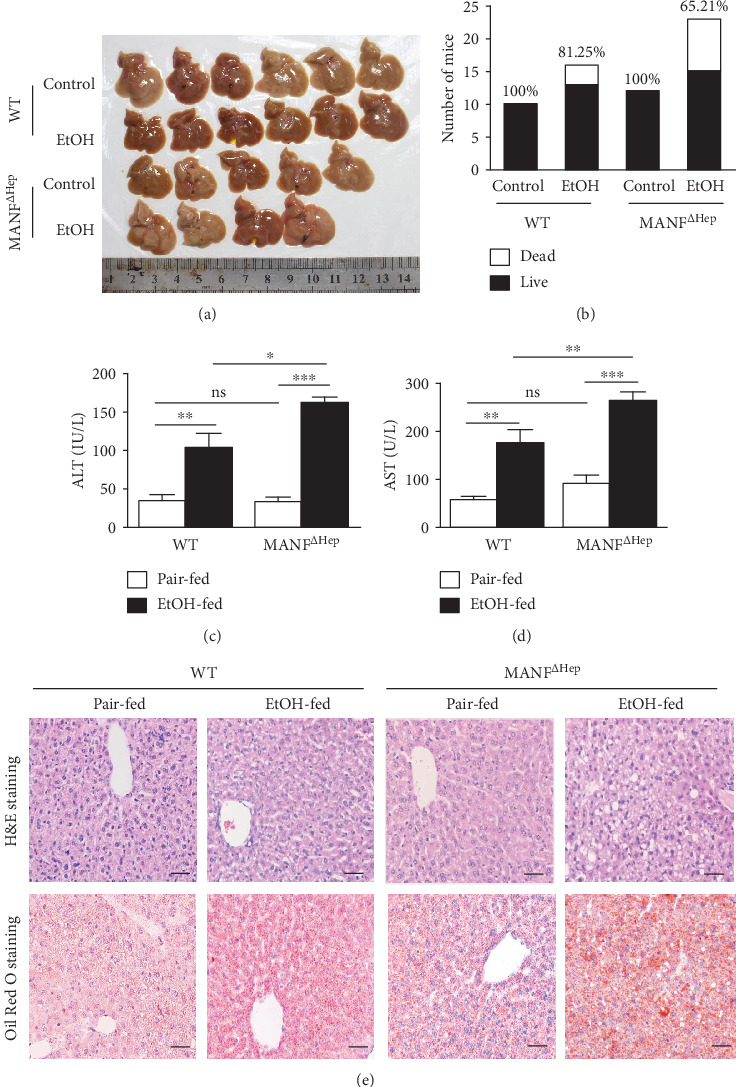
MANF^*Δ*Hep^ mice are more susceptible to EtOH-induced liver injury. (a) Representative images of liver morphology. (b) Survival rate and the number of mice in WT and MANF^*Δ*Hep^ was 26 and 35, respectively. (c) Serum ALT levels of mice. (d) Serum AST levels of mice. (e) Representative images of H&E and Oil Red O staining. Scale bar is 40 *μ*m. Values represent means ± SEM (*n* = 4-8). ^∗^*P* < 0.5, ^∗∗^*P* < 0.01. EtOH: ethanol.

Next, we observed that the livers of MANF^*Δ*Hep^ mice were swollen compared with those of WT mice after ethanol treatment (Figure 2(a)). During the ethanol treatment period, both WT and MANF^*Δ*Hep^ mice had similar food intake and weight change and exhibited normal behaviour. However, MANF^*Δ*Hep^ mice began to exhibit signs of acute illness between 4 and 8 days, characterized by weight loss and decreased activity. These changes were followed by death. The survival rate was determined 9 h after the oral gavage, and the percentage of survival is shown on the top of the bars (Figure 2(b)). The survival rate was 100 percent in both WT and MANF^*Δ*Hep^ mice without ethanol treatment, but it was decreased after ethanol feeding. The survival markedly reduced in ethanol-fed MANF^*Δ*Hep^ mice when compared with WT mice. Chronic-plus-binge alcohol feeding led to increasing the liver-to-body weight ratio in both WT and MANF^*Δ*Hep^ mice, but there was no significant difference in these two groups (Supplementary Figure 1A). Serum ALT and AST levels were elevated after ethanol feeding in both WT and MANF^*Δ*Hep^ mice. Furthermore, MANF^*Δ*Hep^ mice displayed higher levels of serum ALT and AST compared with WT controls after alcohol exposure (Figures 2(c) and 2(d)). Under pair-fed conditions, the evaluated index for liver injury displayed comparable levels between WT and MANF^*Δ*Hep^ mice, except for a slight (not statistically) increase in serum AST in MANF^*Δ*Hep^ mice (Figures 2(c) and 2(d)). Moreover, alcohol exposure increased hepatic triglyceride levels and the increasing levels were comparable between WT and MANF^*Δ*Hep^ mice (Supplementary Figure 1B). We also observed that the hepatic steatosis in MANF^*Δ*Hep^ mice was more severe than that in WT mice, as indicated by H&E staining (Figure 2(e)). Similarly, Oil Red O staining showed significant hepatic fat accumulation in the MANF^*Δ*Hep^ mice, while minor steatosis was found in the WT group after ethanol exposure (Figure 2(e)). MANF^*Δ*Hep^ mice displayed signs of hepatic steatosis and more severe liver injury with increased ALT and AST levels than WT mice after alcohol feeding, suggesting that hepatocyte MANF-deficient mice are more susceptible to ALI.

### 3.3. Hepatocyte MANF Deletion Exacerbates the Accumulation of Inflammatory Cells in the Liver after Ethanol Exposure

**Figure 3:**
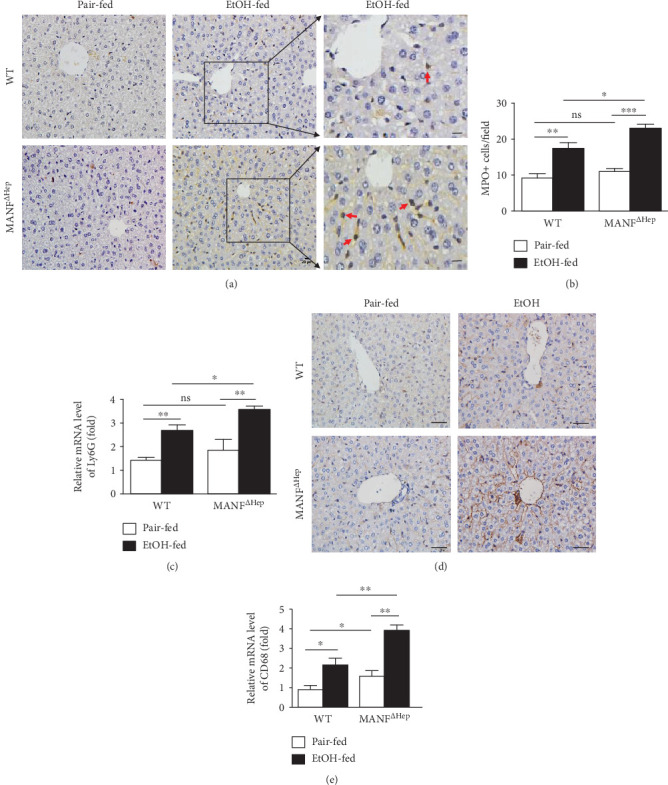
Hepatocyte MANF deletion activates inflammatory cells in alcohol-induced liver injury. (a) MPO^+^ neutrophils were detected by the immunohistochemical assay in WT and MANF^*Δ*Hep^ mice fed with ethanol (EtOH). Scale bar = 20 *μ*m. (b) The quantitative data of panel (a). (c) The mRNA level of Ly6G was detected by quantitative real-time PCR. Values represent means ± SEM (*n* = 4-8). ^∗^*P* < 0.5, ^∗∗^*P* < 0.01, and ^∗∗^*P* < 0.001. (d) Liver tissues were subjected to immunostaining with an anti-CD68 antibody. Scale bar = 40 *μ*m. (e) The mRNA level of CD68 was detected by quantitative real-time PCR. The values were expressed as means ± SEM (*n* = 6–8). ^∗^*P* < 0.05, ^∗∗^*P* < 0.01.

Myeloperoxidase (MPO) and Ly6G (lymphocyte antigen 6 complex, locus G) are commonly used as markers to identify neutrophils [26]. An immunohistochemistry assay showed more MPO-positive cells presented in the liver tissue of MANF^*Δ*Hep^ mice than those in WT mice after ethanol feeding (Figures 3(a) and 3(b)). As illustrated in Figure 3(c), the hepatic expression of Ly6G mRNA was also higher in ethanol-treated MANF^*Δ*Hep^ mice than in ethanol-treated WT mice. These findings suggest that hepatocyte MANF deficiency increased the number of neutrophils in liver tissue of mice treated with alcohol.

Cluster of differentiation 68 (CD68) is a lysosomal membrane protein expressed in macrophages. CD68 is upregulated by proinflammatory stimuli in actively phagocytic cells [27, 28]. Moreover, activation of the CD68^+^ macrophage has also been associated with ALD. Expression of CD68 was evaluated based on immunohistochemistry staining results (Figure 3(d)). Ethanol exposure led to an increased number of CD68^+^ macrophages (which are associated with fibrosis and liver damage) [29] in livers from both MANF^*Δ*Hep^ mice and WT mice. We also observed that the increase in CD68^+^ macrophages was more significant in MANF^*Δ*Hep^ mice than in WT mice. Further, the hepatic expression of CD68 mRNA was elevated in both MANF^*Δ*Hep^ and WT mice after ethanol feeding with much higher levels in MANF^*Δ*Hep^ (Figure 3(e)). Interestingly, this increase in CD68 mRNA was also seen in MANF^*Δ*Hep^ mice compared with WT mice under pair-fed conditions. We also examined the hepatic expression of F4/80 mRNA, but no significant difference was observed in the two groups after ethanol treatment (Supplementary Figure 2).

### 3.4. Hepatocyte MANF Deletion Activates iNOS/NF-*κ*B in Alcohol-Induced Liver Injury

**Figure 4:**
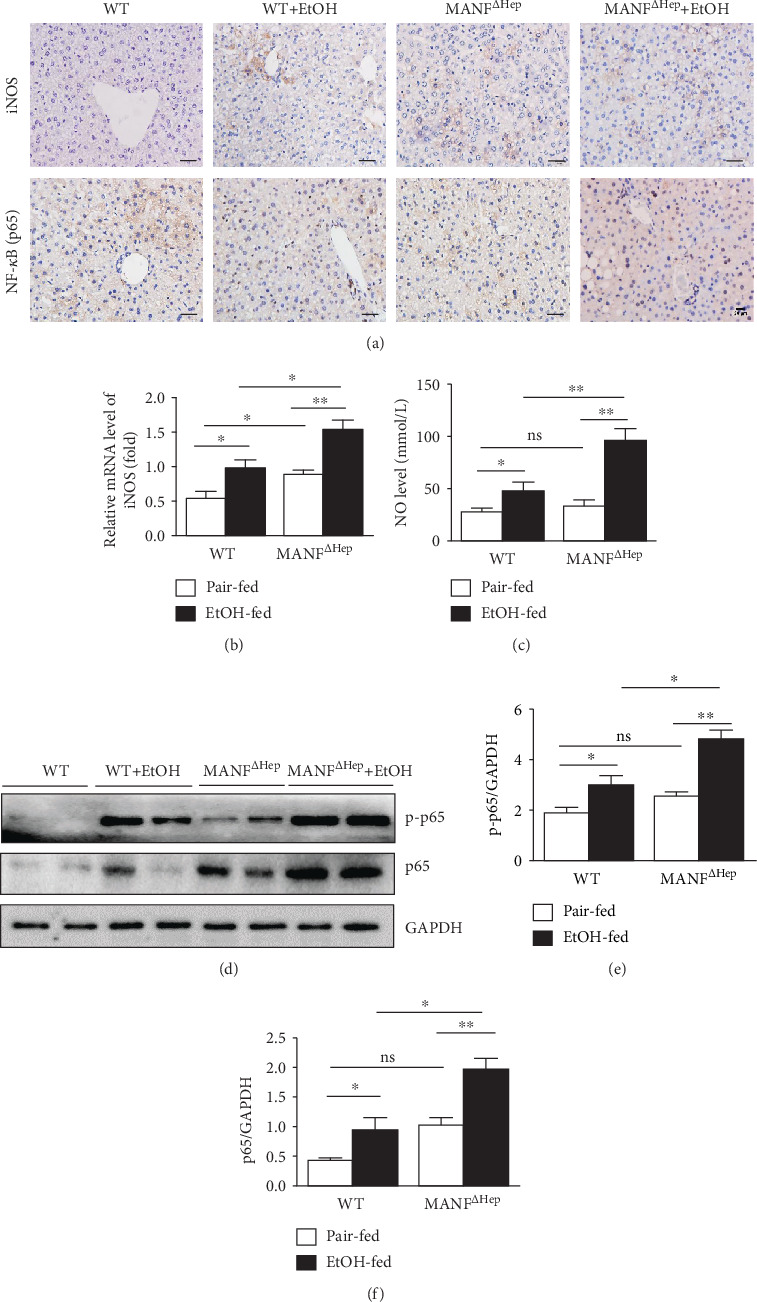
Hepatocyte MANF deletion activates iNOS/NF-*κ*B in alcohol-induced liver injury. (a) Representative iNOS and p65 immunohistochemistry staining in liver tissues. Scale bar = 40 *μ*m. (b) The relative mRNA level of iNOS was detected by using qPCR. (c) Nitric oxide (NO) level was detected with an assay kit. (d) Western blot analysis of hepatic expression levels of p65 and p-p65. (e) The quantitative data of p-p65 in (d). (f) The quantitative data of p65 in (d). The values were expressed as means ± SEM (*n* = 6–8). ^∗^*P* < 0.05, ^∗∗^*P* < 0.01, and ^∗∗∗^*P* < 0.001. p-p65: phosphorylated p65.

The classical inflammatory signaling pathway is the NF-*κ*B signaling pathway. NO produced by iNOS plays an important role in the stimulation of the NF-*κ*B signaling pathway during inflammation. Phosphorylation of the p65 subunit is important for cytoplasmic to nuclear translocation of NF-*κ*B/p65 and induction of transcription of downstream target genes [30]. Therefore, we also investigated iNOS and NF-*κ*B activation in the Gao-Binge model. Immunohistochemistry analysis revealed that the number of iNOS- and p65-positive cells was increased in ethanol-treated MANF^*Δ*Hep^ mice compared with WT mice (Figure 4(a), Supplementary Figure 3A-B). Consistently, the mRNA level of iNOS was significantly increased in MANF^*Δ*Hep^ mice compared with WT mice after alcohol exposure (Figure 4(b)). The mRNA level of iNOS displayed a significant increase in MANF^*Δ*Hep^ mice under pair-fed conditions. Free radical nitric oxide (NO) is primarily produced by iNOS during inflammation. There was an apparent increase in hepatic NO levels in MANF^*Δ*Hep^ mice compared with WT mice after ethanol feeding (Figure 4(c)).

To confirm the above results, we performed western blot to detect NF-*κ*B (p65) protein levels in the liver tissues (Figure 4(d)). We found that the levels of p65 and phosphorylated p65 (p-p65) were increased after ethanol feeding, and there was a statistical difference between WT and MANF^*Δ*Hep^ mice (Figures 4(d)–4(f)). We also noticed that only deficiency of MANF upregulated the levels of p65 and phosphorylated p65 (Figure 4(d)), but the quantitative analysis showed no statistical difference between WT and MANF^*Δ*Hep^ mice (Figures 4(e) and 4(f)).

### 3.5. Hepatocyte MANF Deletion Activates the Downstream Genes of NF-*κ*B in Alcoholic Liver Injury

**Figure 5:**
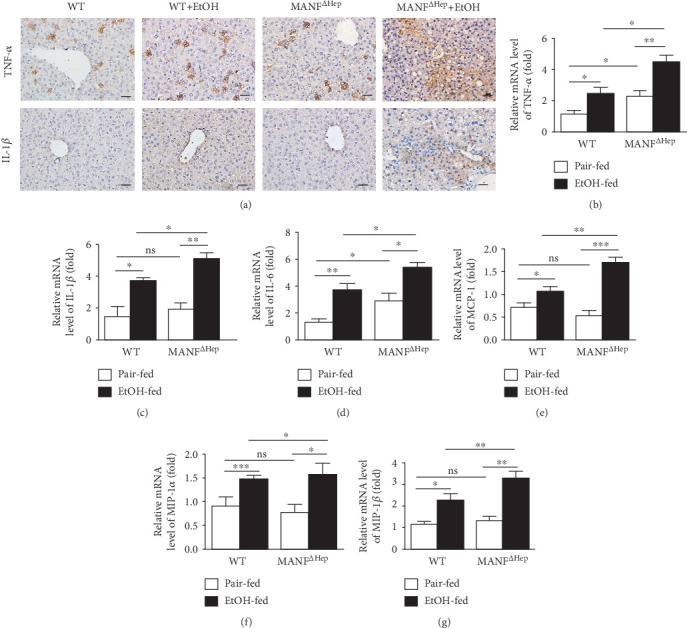
Hepatocyte MANF deletion activates the downstream genes of NF-*κ*B in alcoholic liver injury. (a) Liver tissues were subjected to immunostaining with anti-TNF-*α* and IL-1*β* antibodies. Representative images are shown. Scale bar = 40 *μ*m. (b–g) The relative mRNA levels of TNF-*α* (b), IL-1*β* (c), IL-6 (d), MCP-1 (e), MIP-1*α* (f), and MIP-1*β* (g) were detected by real-time PCR analysis. The values are expressed as means ± SEM (*n* = 6–8). ^∗^*P* < 0.05, ^∗∗^*P* < 0.01, and ^∗∗∗^*P* < 0.001.

Next, we investigated the expression of NF-*κ*B downstream target genes in alcoholic liver injury. The protein levels of TNF-*α* and IL-1*β* were increased in MANF^*Δ*Hep^ mice, compared with WT mice after alcohol feeding by using the immunohistochemical assay (Figure 5(a)). The mRNA levels of TNF-*α*, IL-1*β*, IL-6, monocyte chemoattractant protein-1 (MCP-1), macrophage inflammatory protein-1*α* (MIP-1*α*), and macrophage inflammatory protein-1*β* (MIP-1*β*) were elevated in both MANF^*Δ*Hep^ and WT mice after ethanol feeding. Notably, the increase was more significant in MANF^*Δ*Hep^ mice than in WT mice (Figures 5(b)–5(g)). Under pair-fed conditions, the mRNA levels of IL-1*β*, MCP-1, MIP-1*α*, and MIP-1*β* displayed comparable levels between WT and MANF^*Δ*Hep^ mice, except for a significant increase in TNF-*α* and IL-6 in MANF^*Δ*Hep^ mice. We also found that the expression of IL-1*α* in the alcoholic liver of MANF^*Δ*Hep^ mice was higher than that in WT mice (Supplementary Figure 4). These results indicate that MANF deletion aggravates the production of alcohol-induced inflammatory cytokines, which suggests that MANF has anti-inflammation in alcohol-induced liver injury.

### 3.6. MANF^*Δ*Hep^ Mice Are More Sensitive to Oxidative Stress in Alcoholic Liver Injury

**Figure 6:**
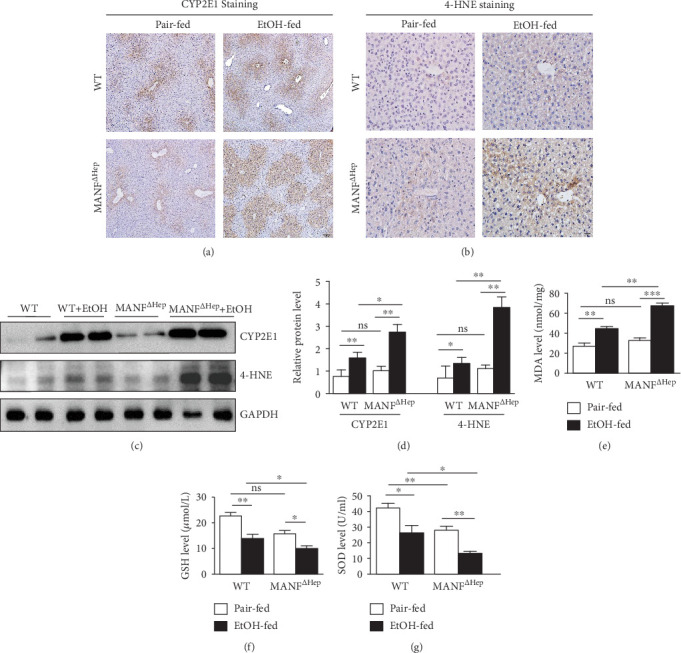
Hepatocyte MANF deletion activates oxidative stress in alcohol-induced liver injury. (a) Expression of hepatic cytochrome P450 2E1 (CYP2E1) in the liver tissues was detected by immunohistochemistry. Scale bar = 100 *μ*m. (b) Expression of 4-HNE in the liver was detected by immunohistochemistry. Scale bar = 20 *μ*m. (c) The protein levels of CYP2E1 and 4-HNE were detected by the western blot method. GAPDH was used as a loading control. (d) The quantitative data in (c). (e–g) The levels of MDA (e) and GSH (f) as well as the activity of SOD (g) in the liver tissues were detected with assay kits. The values were expressed as means ± SEM (*n* = 6–8). ^∗^*P* < 0.05, ^∗∗^*P* < 0.01, and ^∗∗∗^*P* < 0.001.

It was well known that ethanol can induce CYP2E1 expression and activity, which elevates ROS and leads to oxidative stress [31]. Next, we examined whether CYP2E1 was associated with the more severe alcoholic liver injury observed in MANF^*Δ*Hep^ mice. As shown in Figure 6(a), CYP2E1 protein expression was markedly induced after ethanol feeding. Moreover, the increase in CYP2E1 was more prominent in MANF^*Δ*Hep^ mice compared with WT mice. Consistent with IHC results, the protein level of CYP2E1 was confirmed by western blot that indicates that CYP2E1 expression was greatly enhanced by ethanol (Figures 6(c) and 6(d)), and alcohol-induced CYP2E1 expression was more pronounced in MANF^*Δ*Hep^ mice (about 3-fold) than in WT mice (about 2-fold) under pair-fed conditions. 4-Hydroxynonenal (4-HNE) is a stable by-product of lipid peroxidation due to oxidative stress. Results from both immunohistochemistry staining and western blot collectively indicate that the 4-HNE production after ethanol feeding was more substantial in MANF^*Δ*Hep^ mice compared with WT mice (Figures 6(b)–6(d)). In order to further illustrate the impact of hepatocyte-derived MANF on oxidative stress, we next examined additional markers, including malondialdehyde (MDA), GSH, and SOD in the liver tissues. As illustrated in Figures 6(e)–6(g), ethanol increased the production of MDA, the end product of lipid peroxidation with a greater degree in MANF^*Δ*Hep^ mice than in WT mice. In addition, hepatic levels of GSH and SOD, the two essential components in the antioxidant defense system against ROS, were significantly decreased by ethanol exposure, and the observed reduction in MANF^*Δ*Hep^ mice was more pronounced than that in WT mice. The level of SOD also was decreased in MANF^*Δ*Hep^ mice under normal condition as compared to WT mice. However, there was no significant difference between these two groups in MDA and GSH. We also found that the expression of 3-nitrotyrosine (3-NT) in the alcoholic liver of MANF^*Δ*Hep^ mice was higher than that of WT mice (Supplementary Figure 5). These findings indicate that MANF deletion promotes CYP2E1 activation and ROS production in alcohol-induced liver injury, which suggests that MANF may reduce oxidative stress induced by ethanol in the liver.

### 3.7. MANF Deficiency Aggravates Ethanol-Induced Hepatic ER Stress

**Figure 7:**
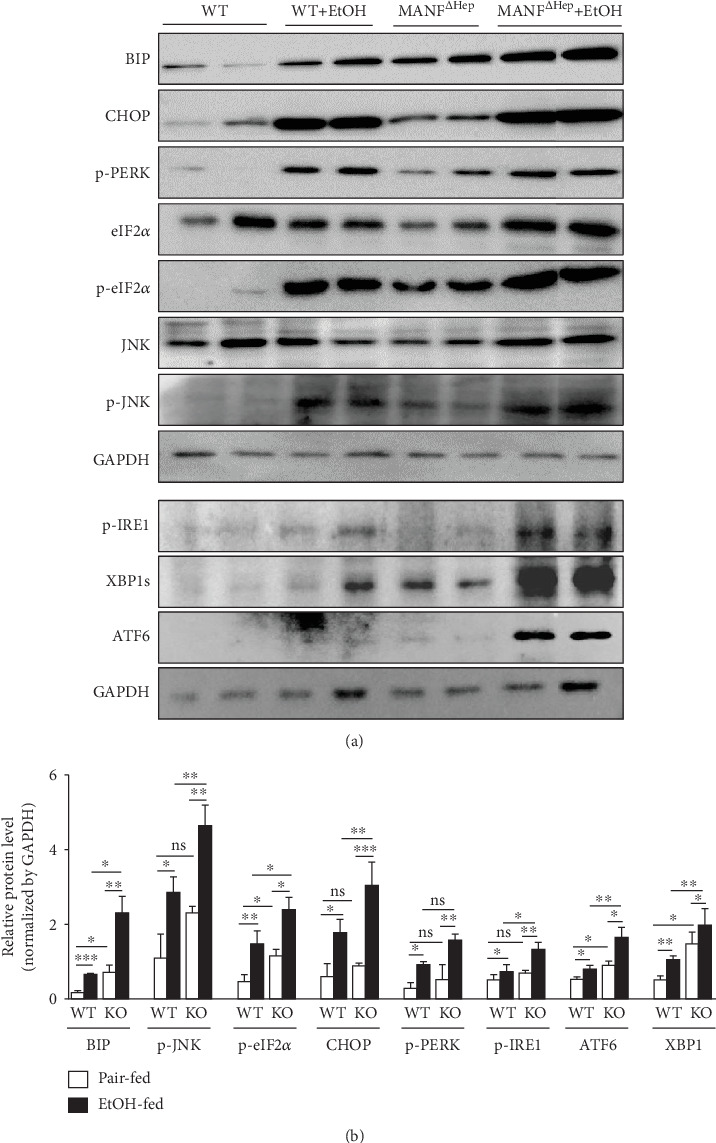
MANF deficiency aggravates ethanol-induced hepatic ER stress. (a) The proteins related to ER stress were detected by western blot. GAPDH was used as a loading control. (b) The quantitative data in (a). The values were expressed as means ± SEM (*n* = 6–8). ^∗^*P* < 0.05, ^∗∗^*P* < 0.01, and ^∗∗∗^*P* < 0.001. WT: wild type; KO: MANF^*Δ*Hep^.

Studies have shown that enhanced ER stress contributes to the pathogenesis of alcohol-induced liver injury [5, 32–34]. MANF is an ER stress-inducible protein. Previous studies found that the cells with high level of MANF exhibited higher tolerance to ER stress-induced cell apoptosis. Therefore, we suspected that the increased susceptibility to alcohol-induced liver injury in MANF^*Δ*Hep^ mice was subject to more severe ER stress in the hepatocyte. We examined the markers of ER stress, BIP and CHOP, and UPR-associated gene expression in the liver tissues. As illustrated in Figures 7(a) and 7(b), BIP and CHOP were upregulated after alcohol feeding, and the upregulation was more significant in MANF^*Δ*Hep^ mice than in WT mice. In MANF^*Δ*Hep^ mice, a more significant increase in hepatic CHOP protein expression was further confirmed by immunostaining, compared with WT mice after treatment with ethanol (Supplementary Figure 6). These results suggest that MANF aggravates ethanol-induced hepatic ER stress. To know which UPR signal was activated, we further detected the UPR genes. We found that phosphorylated eukaryotic initiation factor 2*α* subunit (p-eIF2*α*), phosphorylated Jun-amino-terminal kinase (p-JNK), phosphorylated inositol-requiring enzyme-1 (p-IRE1), spliced form of X-box-binding protein 1 (XBP1s), and activating transcription factor 6 (ATF6) were significantly increased in MANF^*Δ*Hep^ mice treated with ethanol, compared with WT mice. However, phosphorylated PERK (p-PERK) showed no significant difference in these two groups. These results suggest that MANF deficiency activates three UPR signals.

## 4. Discussion

The liver plays a key role in alcohol metabolism and is the primary victim of alcohol-induced injury. Alcoholic liver disease is related to steatosis, inflammation, oxidative stress, and ER stress [3]. Recently, a chronic-plus-single-binge alcohol feeding mouse model is widely used for ALI. This model was able to recapitulate some pathological hallmarks of ALI in ALD patients by mimicking the drinking patterns in humans [19].

In this study, we analyzed hepatic MANF expression in mice with ALI. The results about MANF mRNA and protein levels indicated that MANF expression was upregulated by alcohol. The role of MANF in ALI was further investigated using hepatocyte-specific MANF-knockout mice. We found that MANF^*Δ*Hep^ mice were more susceptible to alcohol-induced liver injury than WT mice. For example, alcohol induced more significant hepatocyte ballooning and higher levels of serum ALT and AST in liver tissues of MANF^*Δ*Hep^ mice than in those of WT mice. In addition, we observed more aggravated inflammatory responses, tissue injury, and increased neutrophil accumulation in the liver of MANF^*Δ*Hep^ mice than in that of WT mice.

Inflammation plays a significant role in the initiation and development of ALD [3]. The activation of NF-*κ*B is associated with the induction of proinflammatory cytokines and other ROS-generating genes like iNOS [35]. NF-*κ*B is one of the central mediators of proinflammatory pathways. NF-*κ*B activation is an essential event causing the liver injury by stimulating the production of inflammatory cytokines along ALD progression. p50 and p65 dimers are the main components of NF-*κ*B, which are inactive in the cytoplasm by binding to the inhibitory protein I*κ*B in unstimulated conditions. With stimuli, such as alcohol exposure, I*κ*B is phosphorylated by I*κ*B kinase and is subsequently degraded. Released NF-*κ*B is then translocated to the nucleus and promotes the transcription of proinflammatory mediators such as TNF-*α* and IL-6 [36–38]. In this study, we investigated the effect of NF-*κ*B activation in alcohol-induced liver injury. NF-*κ*B p65 was greatly induced in MANF^*Δ*Hep^ mice compared with WT mice after ethanol exposure. Our previous study has demonstrated that MANF inhibited NF-*κ*B transcriptional activity by binding to the DNA-binding domain of p65 in the nuclei [39].

Chronic-plus-binge alcohol intake induces the expression and release of a series of proinflammatory cytokines and chemokines [40]. The increase in the number of macrophages was consistent with the upregulation of numerous hepatic macrophage-related cytokines and chemokines like TNF-*α*, IL-1*β*, MCP-1, and MIP-1*α* in ethanol-fed MANF^*Δ*Hep^ mice versus the WT controls. These results suggest that the enhanced monocyte/macrophage and the corresponding cytokine production contribute to the progression of ALI in MANF^*Δ*Hep^ mice. The hepatocyte-macrophage crosstalk might be partly mediated by the transmission of extracellular vesicles (EVs). They are advanced players in cell-to-cell transmission and perform an essential role in stimulating neutrophil infiltration and inflammation in a variety of diseases, including liver diseases. Macrophages can be activated by the stressed hepatocytes through the release of the EVs that contain chemokines, proteins, lipids, and nucleic acids (e.g., mitochondrial DNA) [41–51].

In this study, we found that the degree of ER stress after alcohol feeding was more severe in the MANF^*Δ*Hep^ model than in WT mice. It has been reported that hepatic CYP2E1 protein levels were upregulated after chronic ethanol feeding, and the elevated CYP2E1 intensified hepatic ER stress [52, 53]. Hence, it is plausible to hypothesize that MANF^*Δ*Hep^ mice exposed to ethanol had elevated hepatic CYP2E1 levels, which in turn made the liver more vulnerable to ethanol-induced ER stress. The increases in MDA and 4-HNE levels in the liver of MANF^*Δ*Hep^ mice were greater than those in WT mice after chronic ethanol feeding. We also found that ethanol consumption caused a decrease in antioxidant defence systems (GSH and SOD levels) and the enhancement of oxidative stress in the liver of MANF^*Δ*Hep^ mice.

It is well known that upregulated ER stress is an essential causal factor in ALD [33, 54]. In the pathogenesis of liver diseases, ER stress activated JNK, which performs a vital role. Consistent with these reports, our present study showed that the alcohol intake significantly upregulated the hepatic levels of ER stress marker proteins such as levels of phosphorylated eIF2*α*, JNK, IRE1, ATF6, and XBP1 in the liver of MANF^*Δ*Hep^ mice. These results suggest that hepatic ER stress was activated in the pathogenesis of alcohol-induced liver injury in MANF^*Δ*Hep^ mice. The immunostaining results show a slight increase in CHOP protein in WT mice after alcohol exposure, and this increase became more significant in MANF^*Δ*Hep^ after exposure to alcohol, suggesting that hepatocyte-specific MANF knockout could aggravate alcohol-induced ER stress in MANF^*Δ*Hep^ ethanol-treated mice.

It has also been reported that MANF attenuated the neuronal lesion in MPTP/MPP+-induced Parkinson's disease mice, which might be associated with the inhibition of oxidative stress and the amelioration of mitochondrial function [55]. Recent researches on MANF have concluded that liver-specific MANF ablation in mice caused progressive liver damage, fibrosis, and steatosis. In addition, MANF is protective against age-related metabolic diseases, because it has been established that metabolic stress and age-related damage in a mouse liver were relieved by systematic MANF supplementation [18]. It is also found that MANF prevented cell apoptosis from responding to the injury or disease through the unfolded protein response [12]. These findings suggest that MANF promotes the survival and proliferation of normal cells and protects them against the apoptosis induced by various stimuli.

**Figure 8:**
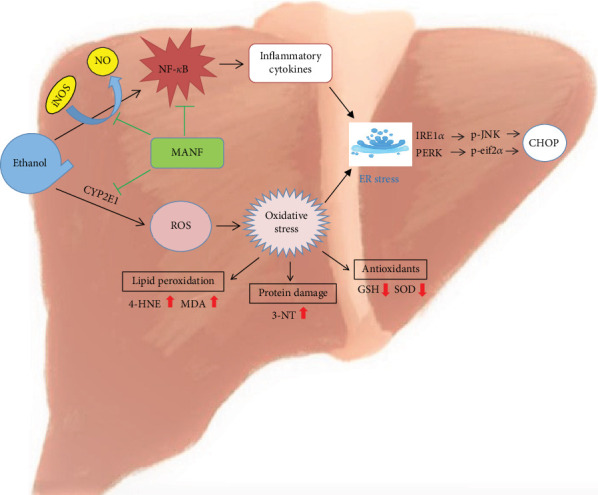
Schematic diagram of the hypothesized mechanisms of MANF's protective role in chronic-plus-binge alcohol feeding.

Mechanistic studies suggested that MANF might ameliorate alcohol-induced liver injury by inhibiting the NF-*κ*B-ROS pathway (see summary in Figure 8). ER stress induced by ethanol plays an essential role in ALD progression. NF-*κ*B is an important transcription factor that plays a major role in regulating the expression of genes related to pathological liver changes. In our study, we found that NF-*κ*B activation induced the expression of a series of proinflammatory cytokines (TNF-*α*, IL-1*β*, and IL-6) and chemokines (MCP-1, MIP-1*α*, and MIP-1*β*) in ethanol-fed MANF^*Δ*Hep^ mice. In addition, alcohol-induced oxidative stress seems to play a significant role in the mechanisms by which alcohol causes liver damage in MANF^*Δ*Hep^ mice. In the livers of MANF^*Δ*Hep^ mice, alcohol consumption develops oxidative stress and ER stress through overproduction of ROS by CYP2E1 and reduced GSH and SOD levels.

Our study demonstrated that MANF, an ER stress-inducible protein, was upregulated by ethanol exposure in WT mice. Hepatocyte-specific MANF deletion in mice increased the severity of steatohepatitis and oxidative stress. Those results suggest that the upregulation of MANF fits in a defensive system in order to maintain the homeostasis throughout an alcohol-induced liver injury response. MANF ameliorated alcohol-induced ER stress by reducing the CYP2E1/oxidative stress signaling pathway and by inhibiting inflammatory response via negative regulation of the NF-*κ*B signaling pathway in the liver. This study suggests that MANF plays a protective role in alcohol-induced liver injury via improvement of ER stress, which is associated with inhibiting iNOS/NF-*κ*B and reducing CYP2E1/oxidative stress signaling pathways. Our findings may shed light on new thoughts for the treatment of ALI.

## Data Availability

The data used to support the findings of this study are available from the corresponding author upon request.

## Abbreviations

ALD: Alcoholic liver disease
ALI: Alcohol-induced liver injury
AST: Aspartate aminotransferase
ALT: Alanine aminotransferase
H&E: Hematoxylin and eosin
MPO: Myeloperoxidase
HNE: 4-Hydroxy-2-nonenal
IL-1*β*: Interleukin 1*β*
TNF-*α*: Tumor necrosis factor *α*
WT: Wild type
MANF^*Δ*Hep^: Hepatocyte-specific MANF knockout.

## Supplementary Materials

Supplementary materials Table 1: primary antibodies for IHC. Table 2: primary antibodies for western blot. Table 3: primer sequences for real-time PCR. Figure 1: (A) liver-to-body weight ratio in WT and MANF^*Δ*Hep^ mice. (B) TG level was examined with an assay kit. Values represent means ± SEM (*n* = 4-8). ^∗^*P* < 0.5, ^∗∗^*P* < 0.01, ^∗∗∗^*P* < 0.001. Figure 2: the mRNA level of F4/80 was detected by quantitative real-time PCR. Figure 3: (A) the quantitative data of iNOS staining. (B) The quantitative data of p65 staining in panel A. Values represent means ± SEM. ^∗^*P* < 0.05, ^∗∗^*P* < 0.01, and ^∗∗∗^*P* < 0.001. Figure 4: detection of IL-1*α* in WT and MANF^*Δ*Hep^ mice. Figure 5: detection of 3-nitrotyrosine in WT and MANF^*Δ*Hep^ mice. Figure 6: detection of CHOP in WT and MANF^*Δ*Hep^ mice.
