# Supplementary material for: Role of Mesencephalic Astrocyte-Derived Neurotrophic Factor in Alcohol-Induced Liver Injury
Source: Oxid Med Cell Longev. 2020 Jul 7;2020:9034864. doi: 10.1155/2020/9034864 (PMC7364207; doi:10.1155/2020/9034864)
Supplement: Supplementary materials — Table 1: primary antibodies for IHC. Table 2: primary antibodies for western blot. Table 3: primer sequences for real-time PCR. Figure 1: (A) liver-to-body weight ratio in WT and MANFΔHep mice. (B) TG level was examined with an assay kit. Values represent means ± SEM (n = 4-8). ∗P < 0.5, ∗∗P < 0.01, ∗∗∗P < 0.001. Figure 2: the mRNA level of F4/80 was detected by quantitative real-time PCR. Figure 3: (A) the quantitative data of iNOS staining. (B) The quantitative data of p65 staining in panel A. Values represent means ± SEM. ∗P < 0.05, ∗∗P < 0.01, and ∗∗∗P < 0.001. Figure 4: detection of IL-1α in WT and MANFΔHep mice. Figure 5: detection of 3-nitrotyrosine in WT and MANFΔHep mice. Figure 6: detection of CHOP in WT and MANFΔHep mice. [file 9034864.f1.docx]

1. **Supplementary Table 1**


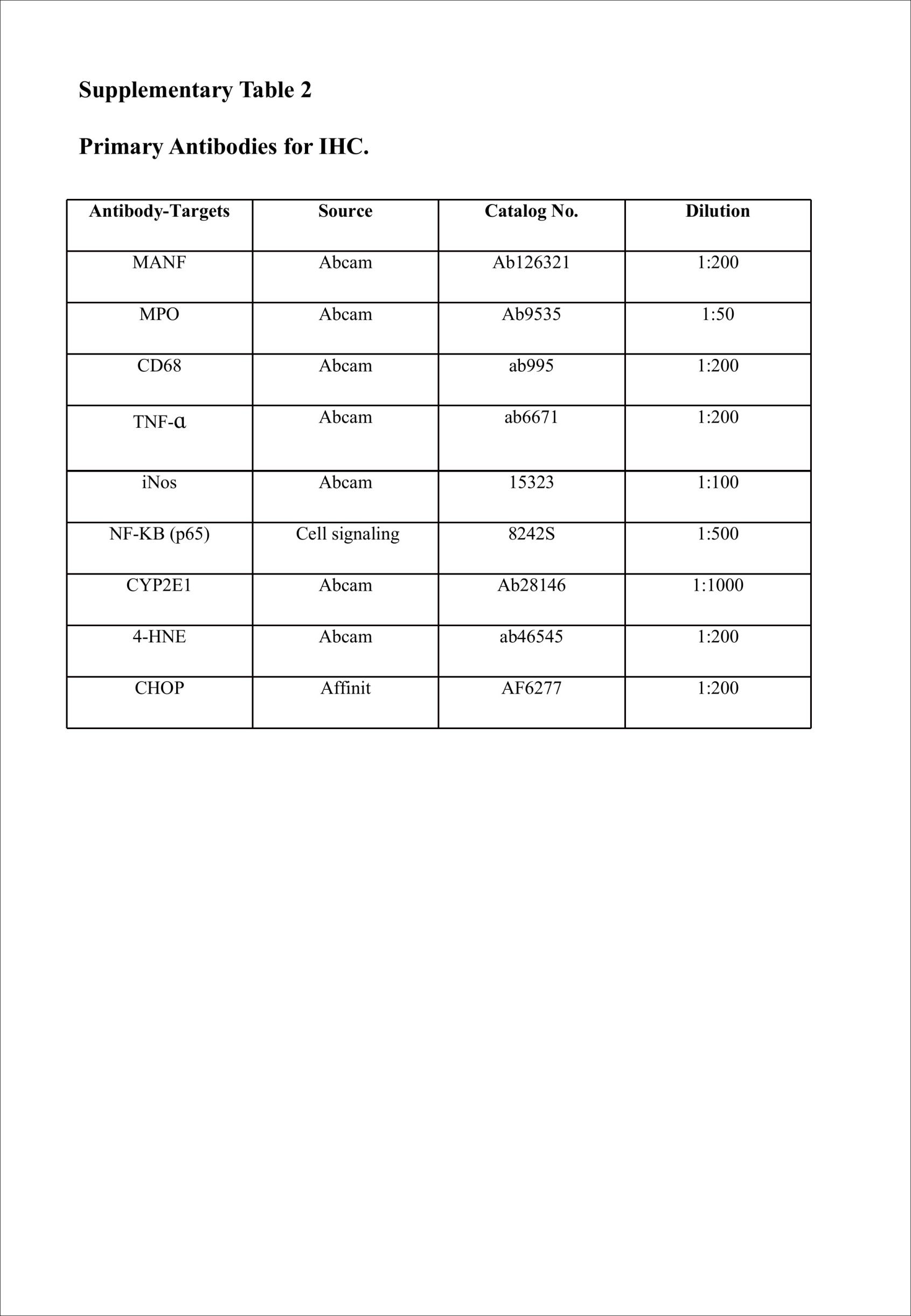


1. **Primary Antibodies for IHC.**
2. **Supplementary Table 2**


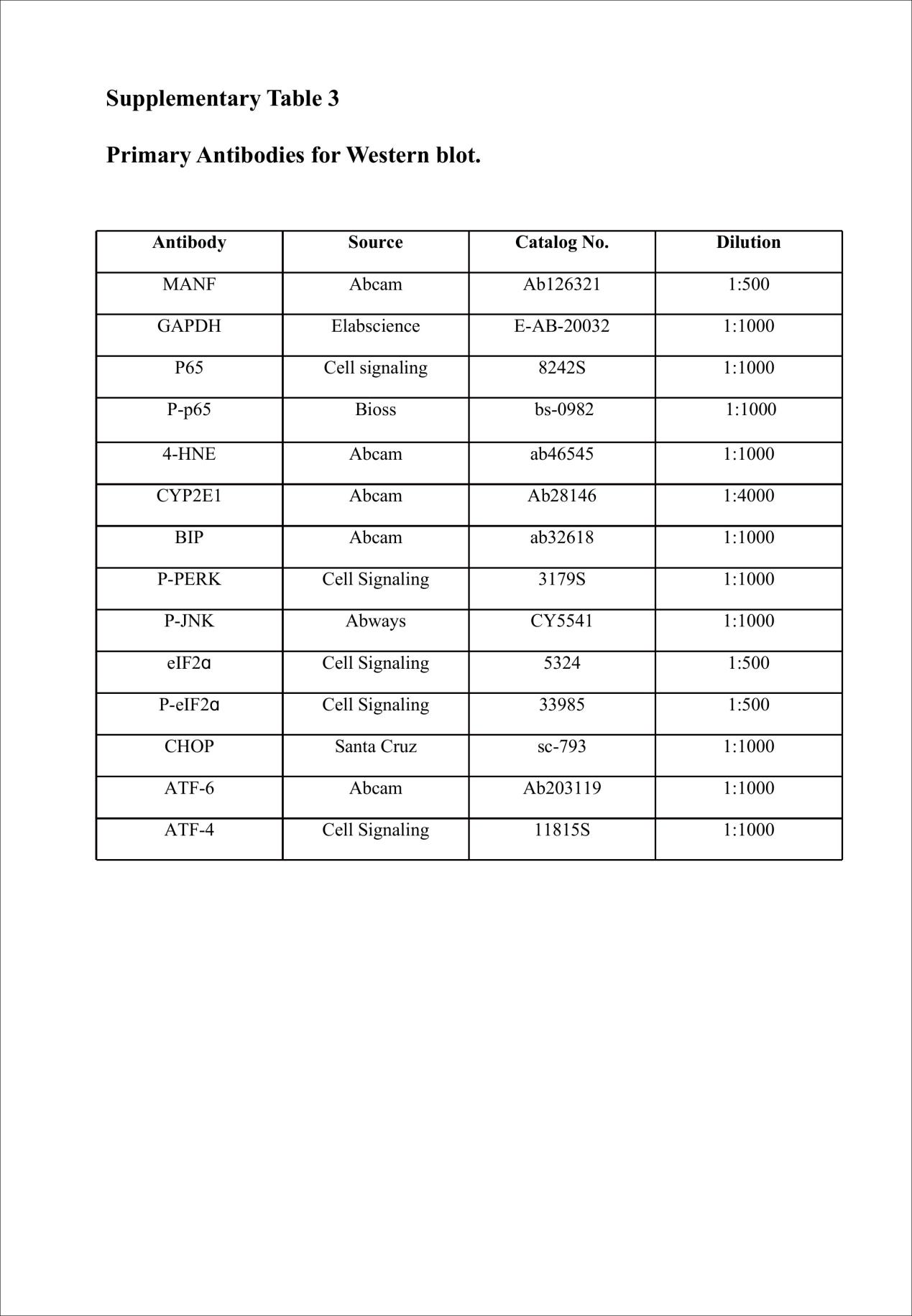


**Primary Antibodies for Western blot.**

1. **Supplemental Table 3**


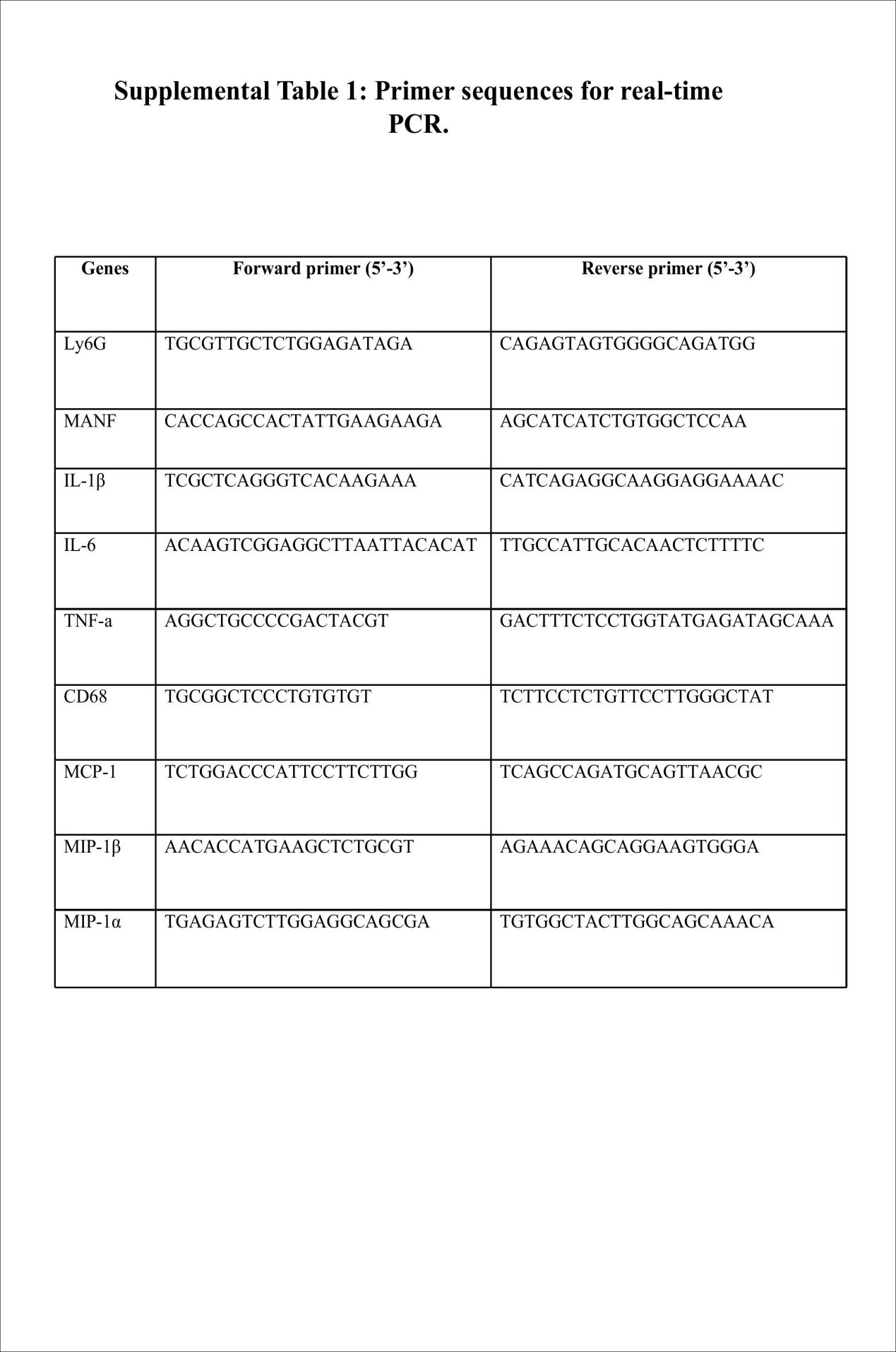


1. **Primer sequences for real-time PCR.**


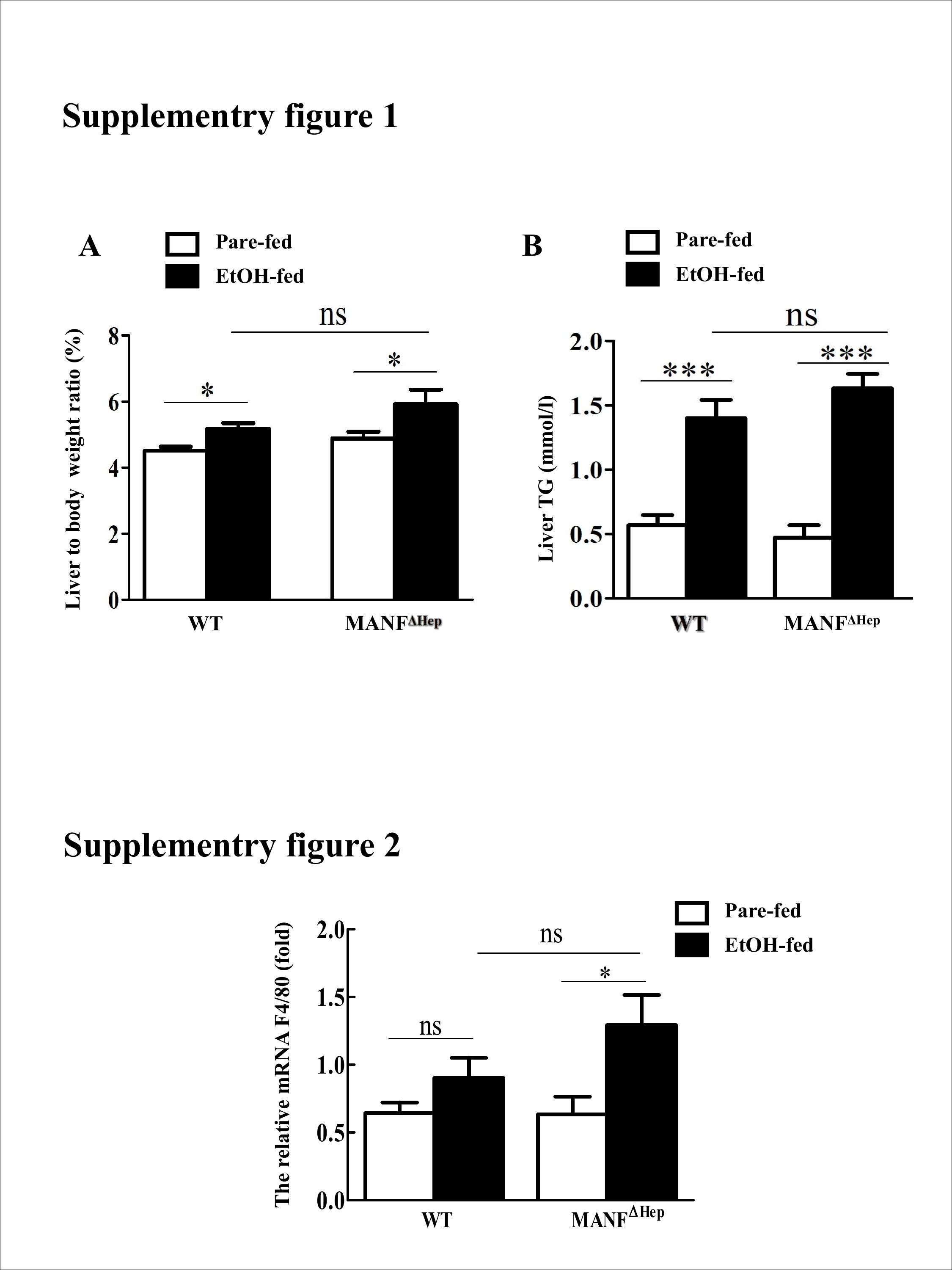


**Supplementary figure 1**

1. liver-to-body weight ratio in WT and MANF^ΔHep^ mice. (B) TG level was examined by an assay kit. Values represent means ± SEM (n= 4-8). *P<0.5, **P<0.01, ***p<0.001.

**Supplementary figure 2**


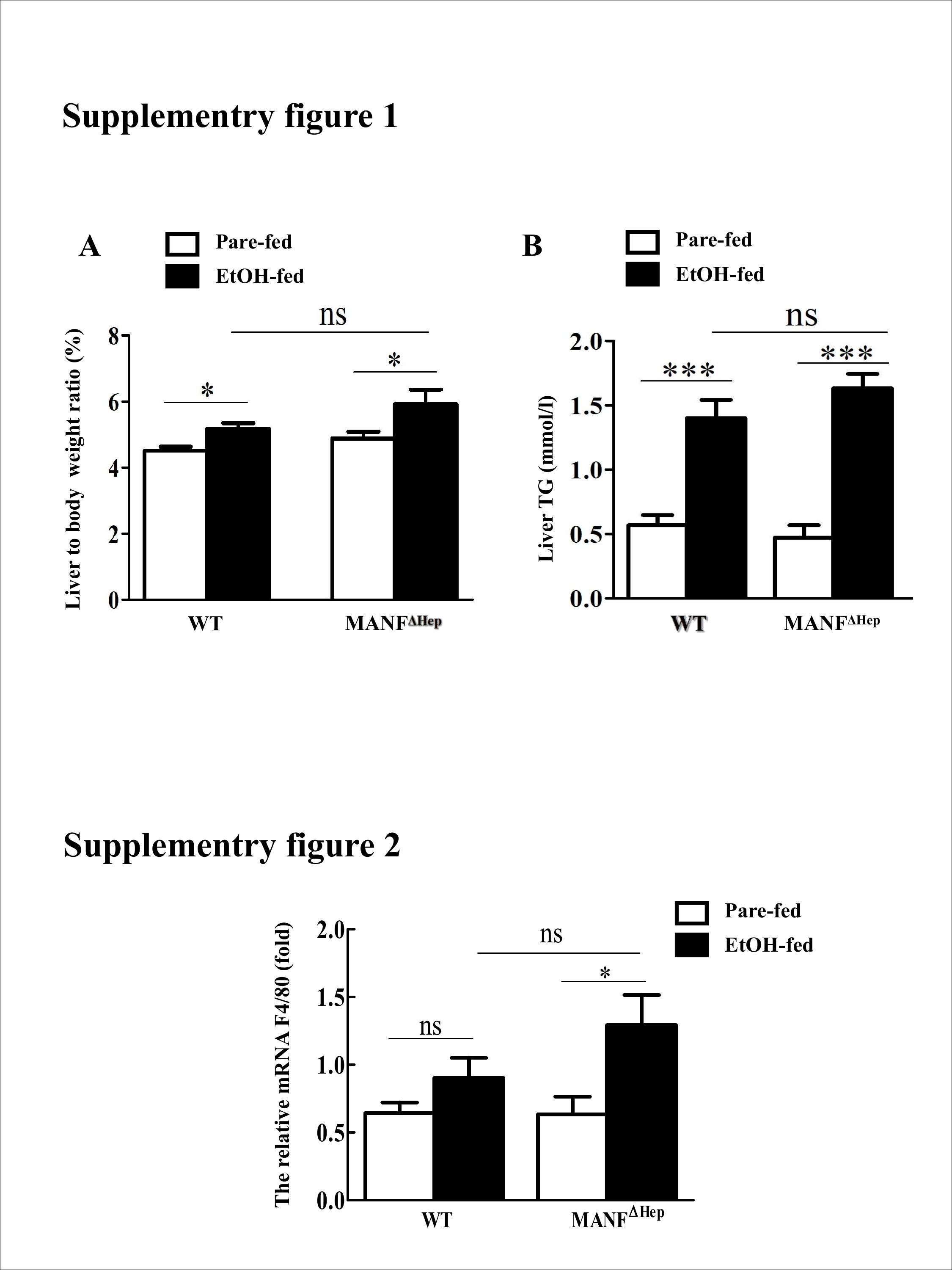


The mRNA level of F4/80 was detected by quantitative real-time PCR. Values represent means ± SEM (n= 4-8). *P<0.5.


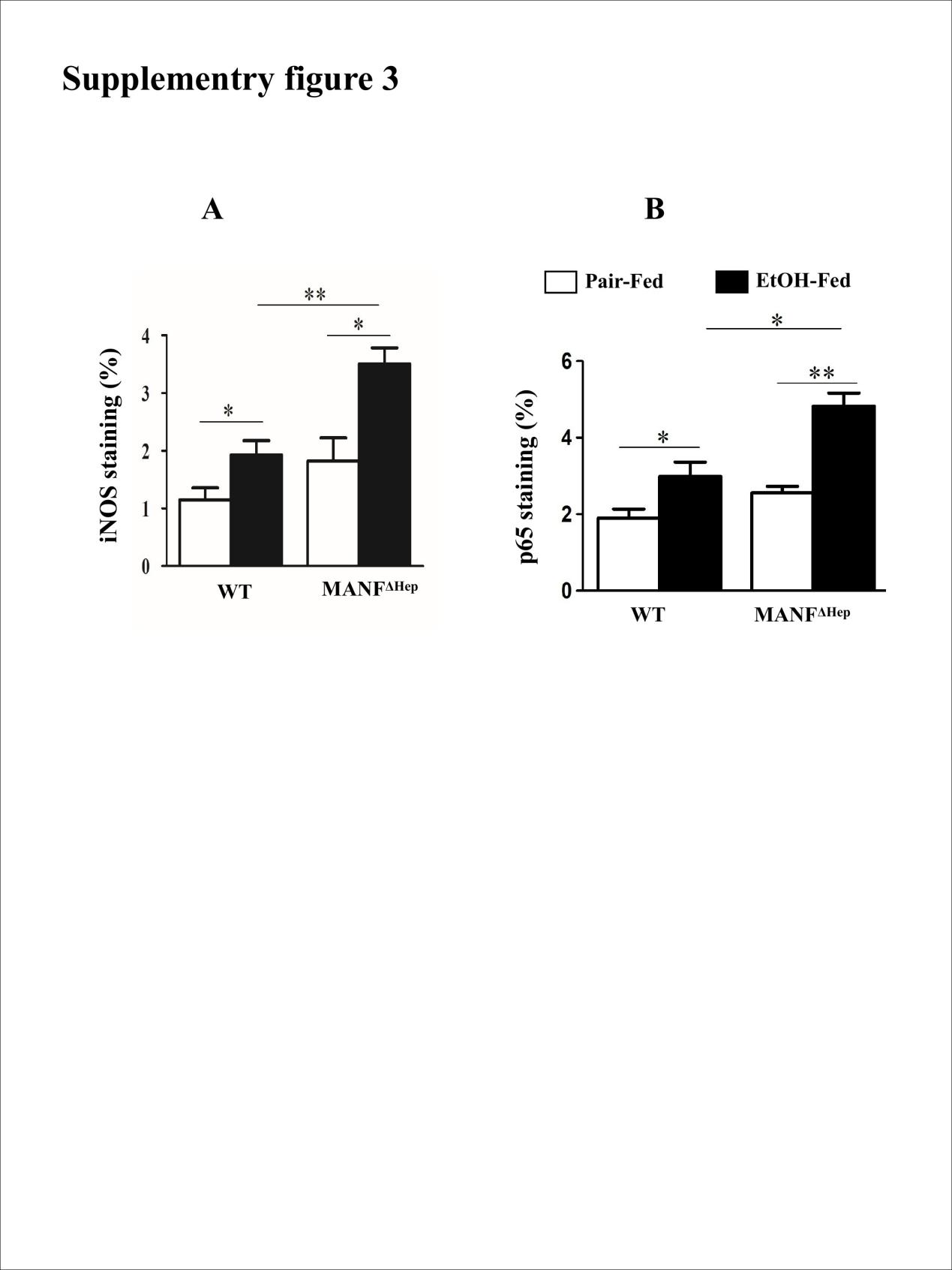


**Supplementary figure 3**

**Supplementry figure 4**

(A) The quantitative data of iNOS staining in panel A. (B) The quantitative data of p65 staining in panel A. . Values represent means±SEM. *p<0.05; **p<0.01; ***p<0.001.

**Supplementary figure 4**


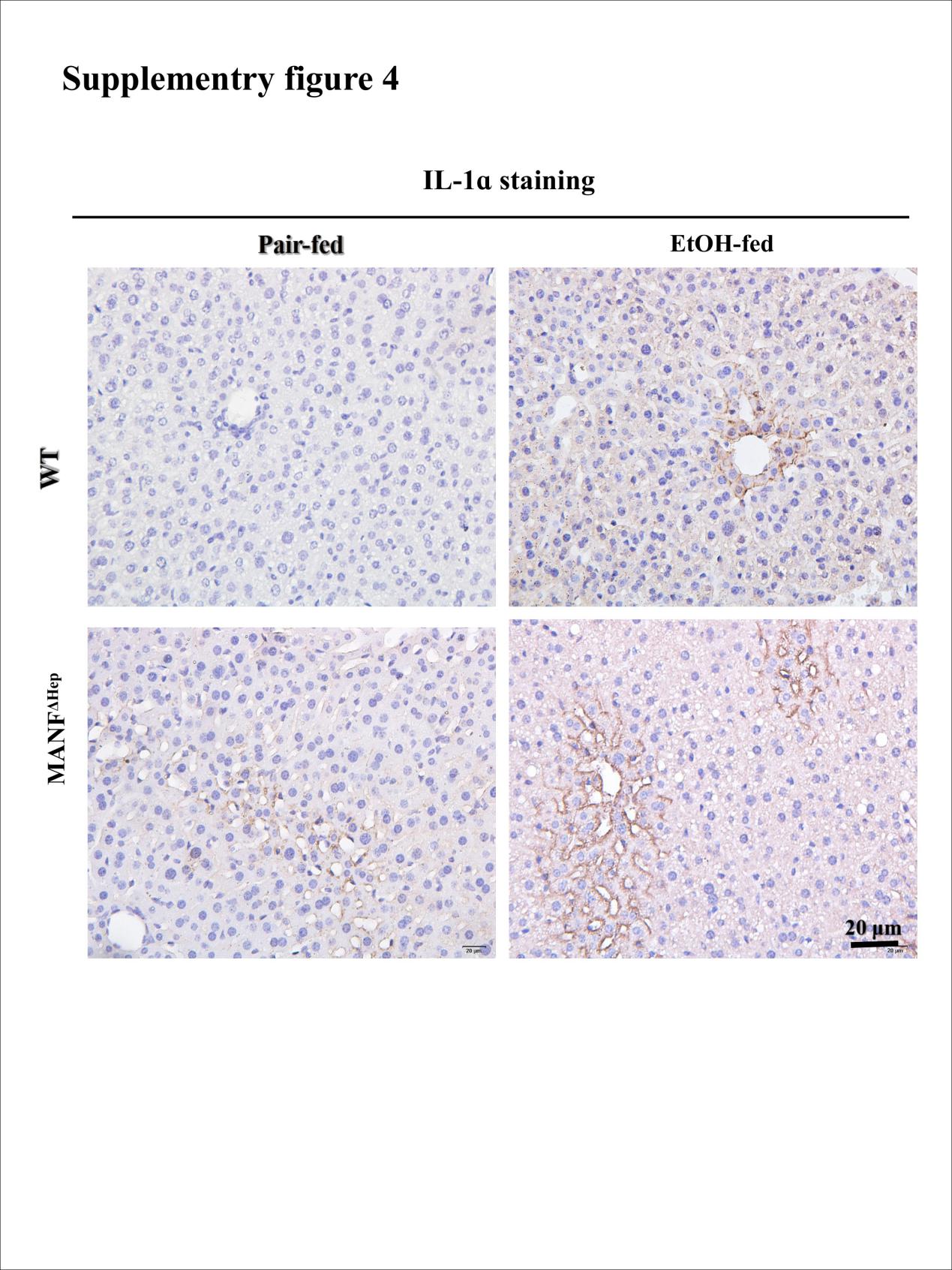


**Figure S4. Detection of IL-1ɑ in WT and MANF^ΔHep^ mice.**

IL-1ɑ^+^ cells were detected by immunohistochemical assay in WT and MANF^ΔHep^ mice fed with ethanol (EtOH). Scale bar=20 μm.

**Supplementary figure 5**


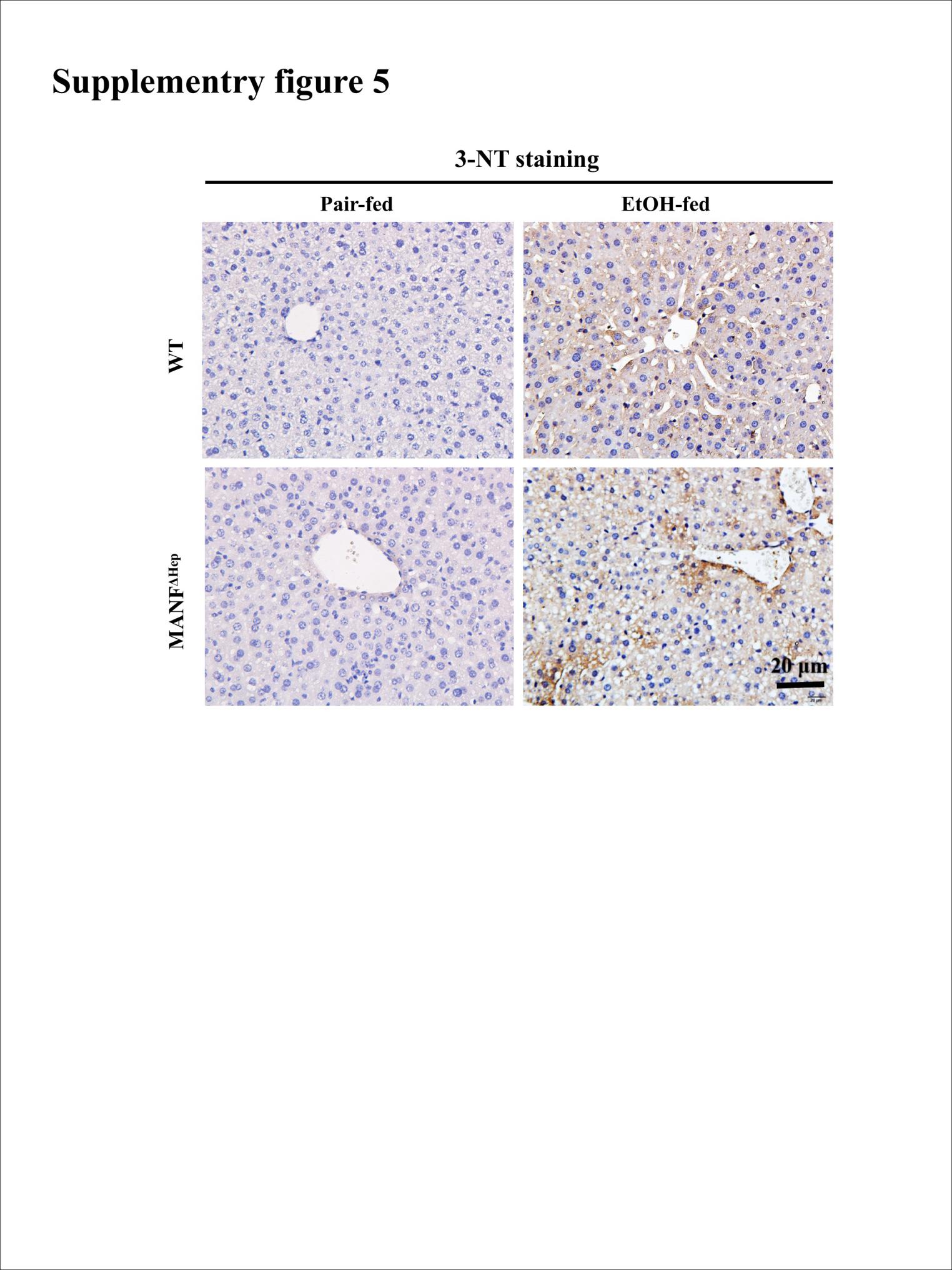


**Figure S5. Detection of 3-nitrotyrosine in WT and MANF^ΔHep^ mice.**

3-NT^+^ cells were detected by immunohistochemical assay in WT and MANF^ΔHep^ mice fed with ethanol (EtOH). Scale bar=20 μm.

**Supplementary figure 6**


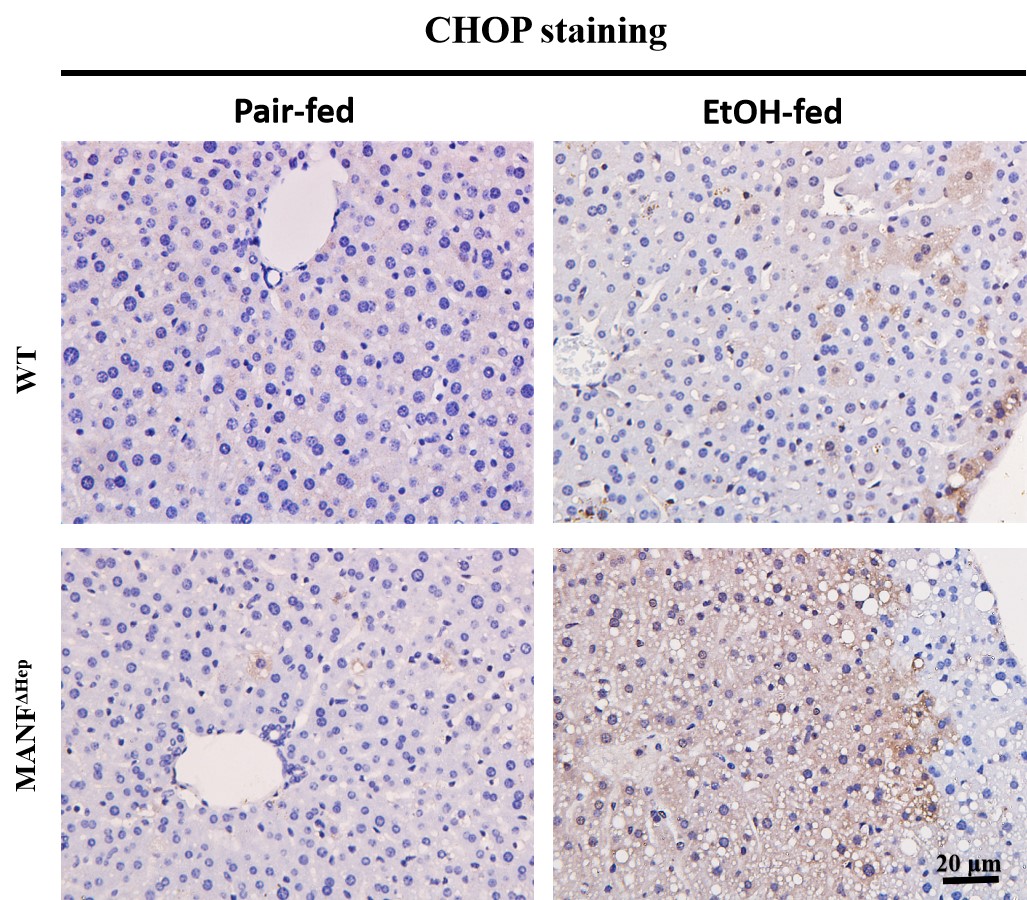


**Figure S6. Detection of CHOP in WT and MANF^ΔHep^ mice.**

CHOP^+^ cells were detected by immunohistochemical assay in WT and MANFΔHep mice fed with ethanol (EtOH). Scale bar=20 μm.
